# Osteogenic Biomaterials in Contemporary Dentistry

**DOI:** 10.1155/2015/945320

**Published:** 2015-04-08

**Authors:** Seong-Hun Kim, Jae-Pyung Ahn, Homayoun H. Zadeh, Eric J. W. Liou

**Affiliations:** ^1^Department of Orthodontics, School of Dentistry, Kyung Hee University, No. 1 Hoegi-dong, Dongdaemun-gu, Seoul 130-701, Republic of Korea; ^2^Advanced Analysis Center, Korea Institute of Science and Technology, 5 Hwarang-ro 14-gil, Seongbuk-gu, Seoul 136-791, Republic of Korea; ^3^Laboratory of Immune Regulation and Tissue Engineering (LITE), Division of Periodontology, Diagnostic Sciences & Dental Hygiene, Ostrow School of Dentistry, University of Southern California, 925 34th Street, Room 4278, Los Angeles, CA 90089-0641, USA; ^4^Department of Orthodontic and Craniofacial Dentistry, The Chang Gung University, Taipei, Taiwan

There have been developments and applications of osteogenic biomaterial substitutes in dentistry to replace missing dentition or to reinforce existing dentitions. The dentistry in practice has evolved into new treatment modality with the development and application of novel biocompatible materials. The examples are dental implants, bone graft materials, surgical plates, and any modification to increase biocompatibility and stability in dentition. These materials cover everything from replacing the missing teeth and/or degenerated supporting structures to the induction of new bone formation. Also, the osseointegrated materials further allowed orthopedic force application on these materials as skeletal anchorage to control tooth movements. Currently, modifications of the surface treatment or combination of osseoinductive materials to improve potential osseointegration are continuously endeavored.

This special issue delivers original research on different aspects, studies on the application of the novel biomaterials to fill bone defect rapidly with new bone, improvement of osseointegration through variety of surface treatment, and the possibility of alumina toughened zirconia for the new implant material, and also covers clinical research on a modified dental implant as a gateway to the human body: implant mediated drug delivery system.

First, we discuss the studies of novel biomaterial application for new bone formation in bony defect. Rapid bone defect filling with normal bone is a challenge in orthopaedics and dentistry. A study by S. Ansari et al. developed a strategy for bone tissue engineering that entails application of immobilized anti-BMP-2 monoclonal antibodies (mAbs) to capture endogenous BMPs in vivo and promote antibody-mediated osseous regeneration (AMOR). These data have potential implications for the mechanism of action of AMOR, suggesting that anti-BMP-2 may capture endogenous osteogenic BMPs, which may in turn mediate de novo bone formation. Strontium ranelate (SrRan) has been shown to in vitro decrease bone resorption and increase bone formation and represents a potential agent with the capacity to accelerate bone defect filling.

G. Zacchetti et al.'s study demonstrates that Sr is integrated both in cortical and in trabecular bone healing of the defect in SrRan-treated rats and improves the bone material level properties of the healing bone mainly after 4 weeks of treatment. These results open up new perspectives for the use of SrRan in clinical studies as a pharmacologic agent with a potential beneficial effect on bone defect repair.

A. Monje et al.'s comprehensive systematic review aimed at assessing the feasibility of allogeneic block grafts by means of survival rate, histologic analysis, and causes of failure, for augmentation of the atrophic maxilla, provides a current state of the art about this treatment modality. Therefore, this study can be of a very meaningful importance for clinicians in the decision making of selecting the ideal source for bone block grafting.

This special issue illuminates the clinical evaluation of the bone graft materials that have been used conventionally and introduces the histological identification of changes in tissue adjacent to the biomaterials through three original research articles. Particularly, clinical experiment on the management of the thin alveolus which has been considered as tooth movement limitation in orthodontics and the scientific analysis of the methods have been discussed in depth.

Alveolar augmented corticotomy is effective in accelerating orthodontic tooth movement, but the effect only lasts for a relatively short time. A study by D.-Y. Lee et al. demonstrated the stable results in a long-term (12 weeks) experiment using xenograft materials in beagle experiment. Absorbable collagen membrane could control the texture of bone surface.

K.-B. Lee et al. researched the different types of bone graft materials on the treatment effect of augmented corticotomy. The remarkable thing is that not only allograft or xenograft but also synthetic graft material showed comparable new bone formation. Augmented corticotomy showed stable results regardless of the graft material types, and in particular synthetic bone material showed dramatic new bone formation.

The study of H.-J. Choi et al. was to investigate the underlying biology of the immediate periodontal response to orthodontic tooth movement after the augmented corticotomy with alloplastic bone grafts. The results demonstrated that measurable tooth movement began as early as 3 days after the intervention in beagle dogs. Based on the results and histological findings, augmented corticotomy-facilitated orthodontic tooth movement might enhance the condition of the periodontal tissue and the stability of the outcomes of orthodontic treatment.

There has been increased interest in search of the new biomaterial for dental implant. We would like to examine the possibility of alumina toughened zirconia for the new implant material through several articles.

S. Y. Kim et al.'s study classified the influence of different topographies and hydrophilicities of Ti surfaces on the expression of various functional factors in PDLSCs involved in osteogenesis in the absence of osteogenic supplements and evaluated biomarkers of cellular activity, including the expression of transcription factors and signaling molecules of PDLSCs on the Ti surfaces.

The relevance of J. Markhoff et al.'s research was to develop a glass solder matrix for coating zirconia ceramics to improve osseointegration of ceramic dental implants with adequate mechanical properties at once. Thereby, the existing advantages in esthetic appearance and hypersensitivity of ceramic dental implants in contrast to metallic implants may become effective in clinical application in the near future.

The results of R. Olivares-Navarrete et al.'s study indicate that effects of the complex SLA topography are greater than the effect of acid etching or grit blasted on regulation of osteogenesis, osteoclastogenesis, and angiogenesis on multipotent BMCs and committed osteoblasts. 1*α*,25(OH)2D3 had a major role in enhancing these effects that was sex depended.

Alumina-zirconia composites have attracted significant interest in the past few years for orthopedic and dental use. Although yttria stabilized zirconia is the only ceramic currently employed for dental implant fabrication, several reasons suggest that alumina-zirconia composites may conveniently substitute this monolithic material. Here, F. Mussano et al. report the results of the experiments performed in vitro and in vivo to assess the behavior of alumina toughened zirconia (ATZ) dental implants. It is important to underline that ATZ is different from zirconia-toughened alumina (ZTA) due to the relative percentage of the two materials and the consequent mechanical and possibly biological properties.

Another topic enlightened in this special issue is dental implant. It is about neither improving osseointegration potential nor improving functional occlusion of implant, but it is a preliminary study on the prospect of dental implant as a drug delivery system. In an article by Y.-S. Park et al., they developed and proposed a new implant mediated drug delivery system (IMDDS) through a modified titanium implant. In the nearest future, the IMDDS may provide a novel approach to treat patients with chronic diseases.

In conclusion, we debated about the current status of osteogenic biomaterials in contemporary dentistry, the osteogenic biomaterial types, the application of the material, and the future study. We would like to suggest a right direction of osteogenic biomaterial in this special issue because comprehension of recent advances in biomaterial of dentistry would lead to appropriate applications of these biomaterials and successful strategies to improve treatment outcomes to better serve patients.

*Seong-Hun Kim* *Jae-Pyung Ahn* *Homayoun H. Zadeh* *Eric J. W. Liou*

